# Impacts of perinatal dioxin exposure on gaze behavior in 2-year-old children in the largest dioxin-contaminated area in Vietnam

**DOI:** 10.1038/s41598-023-47893-0

**Published:** 2023-11-24

**Authors:** Pham Ngoc Thao, Muneko Nishijo, Pham The Tai, Tran Ngoc Nghi, Vu Thi Hoa, Tran Hai Anh, Tran Viet Tien, Yoshikazu Nishino, Hisao Nishijo

**Affiliations:** 1 Department of Functional Diagnosis, 103 Military Hospital, Vietnam Military Medical University, 160 Phung Hung, Ha Dong, 12108 Ha Noi, Vietnam; 2 Department of Functional Diagnosis, 103 Military Hospital, Vietnam Military Medical University, 261 Phung Hung Street, Phuc La Commune, Ha Dong District, Ha Noi, Vietnam; 3 Department of Epidemiology and Public Health, Kanazawa Medical University, Ishikawa, 920-0293 Japan; 4 Institute of Biomedicine and Pharmacy, Vietnam Military Medical University, 12108 Ha Noi, Vietnam; 5 Ministry of Health, Vietnamese Government, Hanoi, Vietnam; 6 Department of Physiology, Vietnam Military Medical University, 12108 Ha Noi, Vietnam; 7 Department of Tropical and Infectious diseases, 103 Military Hospital, Vietnam Military Medical University, 12108 Ha Noi, Vietnam; 8 Department of Sport and Health Sciences, Faculty of Human Sciences, University of East Asia, Shimonoseki-Shi, Yamaguchi, 751-8503 Japan

**Keywords:** Environmental sciences, Health occupations, Neurology

## Abstract

Fifty-five children aged 2 years from a birth cohort in the largest dioxin-contaminated area in Bien Hoa city, Vietnam participated in this survey to examine gaze behavior. Exposure levels were indicated by 2,3,7,8-tetrachlorodibenzo-p-dibenzodioxin (TCDD) and toxic equivalent of polychlorinated dibenzo-p-dioxin and polychlorinated dibenzofuran (TEQ-PCDD/Fs) levels in maternal breast milk. The percentage of the total fixation duration on the face (% Face), mouth (% Mouth), and eye areas (% Eyes) when viewing silent and conversation scenes was used as gaze behavior indices. When they reached 3-year-old, autistic behavior was assessed using the Autism Spectrum Rating Scale (ASRS). A general linear model adjusted for confounding factors was used to compare gaze indices and ASRS scores between high and low dioxin exposure groups. Effects of perinatal dioxin exposure on gaze behavior were found only when viewing conversation scenes indicated by lower % Face for boys in high TCDD exposure group and lower % Eyes for girls in high TEQ-PCDD/Fs group. Increased autistic traits showed by higher ASRS scores at 3-year-old were found in both gender in the high TCDD exposure group. These findings indicate that perinatal TCDD and TEQ-PCDD/Fs exposure may reduce gaze behavior in 2-year-old children, predicting increased autistic traits at 3-year-old.

## Introduction

Bien Hoa airbase is the largest dioxin-contaminated area in Vietnam because of the use of herbicide, particularly Agent Orange. Agent Orange contains 2,3,7,8-tetrachlorinated-p-dibenzodioxin (TCDD), and was used by the US army from 1961 to 1970 in Vietnam. In 2012, we recruited 210 mother–newborn pairs living in 10 communes close to Bien Hoa airbase (Bien Hoa birth cohort 2012) and reported dioxin levels in maternal breast milk^1^. The mean concentrations of TCDD and toxic equivalent (TEQ) of polychlorinated dibenzodioxins and polychlorinated dibenzofurans (PCDD/Fs) in breast milk were 2.6 pg/g lipid and 10.5 pg-TEQ/g lipid for primiparous mothers, and 2.2 pg/g lipid and 9.0 pg-TEQ/g lipid for multiparous mothers, respectively. TCDD levels were nearly four to five times higher in primiparous mothers and seven times higher in multiparous mothers compared with those in unsprayed areas. In 2015, we additionally collected 78 mother–child pairs as the second birth cohort in the Bien Hoa region (Bien Hos cohort 2015). We combined the two groups and reported the effects of perinatal dioxin exposure on expressive language in boys at 2 years old^2^.

In newborn infants from the Bien Hoa birth cohort 2015, we recorded neonatal electroencephalography (EEG) during sleep time on the second day after birth and reported that dioxin exposure, particularly TCDD exposure, altered relative EEG power and coherence in the active sleep stage^3^. We also reported decreased relative EEG power in the quiet sleep stage associated with increasing TCDD exposure, which was leading to poor gaze behavior at 2 years old as shown by a shorter fixation duration on the face of a child talking in videos^4^. However, we didn’t investigate direct effects of dioxin exposure on gaze behavior in these 2-year-old children from Bien Hoa cohort.

We followed up the children at 3 years old in Bien Hoa cohort 2012 and found that increased TCDD exposure decreased the percentage of face fixation duration when viewing face static pictures in girls, which associated with poorer social communication ability examined by the Autism Spectrum Rating Scale^5^. In addition, previous reports have shown a shorter fixation duration on facial areas including eye and mouth areas during dynamic stimuli of viewing a video in children with autism compared with typical children^6,7^. These results suggest that poor gaze behavior at 2 years of age may be a good predictor for increased autistic traits which can be diagnosed at 3 years of age in perinatally dioxin-exposed children in a dioxin hot spot in Vietnam.

Therefore, we firstly investigated direct associations between perinatal dioxin exposure and gaze behavior indicated by fixation duration of eye, mouth, and whole face areas at 2 years old. And then, we investigated the neurodevelopmental status at 3 years old, including general neurodevelopment and autistic traits, in highly exposed children who showed poor gaze behavior at 2 years old compared with lower exposed children.

## Methods

### Subjects and location

Bien Hoa airbase is considered as the most severely dioxin contaminated areas in Vietnam. The Office of the Vietnam National Steering Committee 33 and Hatfield Consultants (2011) reported TCDD soil levels as high as 61,400 pg/g dry weight and TEQ-PCDD/Fs in sediment as high as 5970 pg/g dry weight in samples collected from the Bien Hoa airbase. The contribution of TCDD to these TEQ-PCDD/Fs was more than 80% in most samples^8^.

Seventy-eight mother–newborn pairs living in 10 communes close to Bien Hoa airbase were enrolled in the present study from August to December in 2015. The mother–newborn pairs were included if they met the following criteria: (i) mothers had resided in the target area for at least 1 year before giving birth; (ii) newborns were born at full-term, and (iii) there were no complications during giving birth. At that time, breast milk sample was collected from 67 mothers for dioxin measurement. There were no breast milk samples or not enough breast milk samples for dioxin measurement were found in eleven mothers. In December 2017 and January 2018, we carried out a 2-year follow-up survey in which 66 mother–child pairs (84.6%) participated in an examination of gaze behavior and neurodevelopment^4^. Twelve pairs did not participate in the survey for the following reasons. One mother stayed in hospital, three refused to participate in the survey, five moved to other locations, and three were parents who were too busy with work. Additionally, six children who were not able to achieve successful calibration in the gaze examination or their total fixation duration for all stimuli was < 2.7 s (< 5th percentile), and five children who had missing data of exposure markers or gestational weeks at birth were excluded. Finally, data of 55 children (24 boys and 31 girls) were available for analysis of associations between perinatal dioxin exposure and gaze behavior. There were no significant differences in dioxin concentrations between participants and non-participants (Supple. Data 1). The characteristics of the mothers and families of children, gestational weeks and body size of children at birth, and age and body size of children at the survey are shown in Table 1. Only maternal education was significantly higher in girls compared with boys.

**Table 1:** Comparisons of characteristics of mothers, family, and children and dioxin exposure levels between sexes of children.

Characteristics	Units	All (N = 55)		Boys (N = 24)		Girls (N = 31)		*p*-value
		Mean, [N]	SD, (%)	Mean, [N]	SD, (%)	Mean, [N]	SD, (%)	
Mothers								
Age	Years	29.8	6.3	30.2	7.6	29.4	5.1	0.656
Parity categories (% primiparae)	%	[26]	(47.3)	[11]	(45.8)	[15]	(48.4)	1.000
Education	Year	12.6	3.2	11.6	3.1	13.4	3.0	0.038
Family								
Income per month*	× 10^6^ VND	13.1	4.5	14.1	5.1	12.4	3.8	0.187
Smoking (yes/no)		[30]	(54.5)	[15]	(62.5)	[15]	(48.4)	0.414
Children								
At birth								
Gestational weeks	Weeks	39.2	0.9	39.3	0.7	39.1	1.0	0.408
Weight	g	3312	363	3343	349	3288	377	0.582
Length	cm	50.8	1.4	51.0	1.1	50.6	1.6	0.208
Head circumference	cm	34.3	1.2	34.3	1.3	34.3	1.3	0.971
Abdominal circumference	cm	35.0	1.4	35.2	0.9	34.8	1.7	0.251
At the survey								
Age	months	25.8	0.7	25.7	0.7	25.9	0.7	0.358
Weight*	kg	12.4	1.6	12.9	1.8	12.0	1.4	0.039
Length*	cm	86.3	3.1	87.3	3.4	85.6	2.7	0.046
Head circumference*	cm	46.9	2.1	47.7	1.6	46.3	2.4	0.017
Abdominal circumference*	cm	47.7	3.6	48.1	4.1	47.4	3.2	0.473

Participants		All (N = 50)		Boys (N = 21)		Girls (N = 29)		*p*-value
At the follow-up survey								
Age at the survey	Months	38.7	1.6	38.6	1.7	38.8	1.6	0.620
Weight	kg	15.4	2.7	16.2	2.8	14.8	2.6	0.073
Length	cm	95.4	3.8	96.6	3.8	94.5	3.7	0.055
Head circumference	cm	48.6	1.4	49.3	1.4	48.2	1.2	0.005
Abdominal circumference	cm	49.7	5.8	50.1	6.6	49.7	5.0	0.792
Dioxins in breast milk**								
TCDD	pg/g lipid	2.0	2.1	1.8	2.4	2.2	1.9	0.368
TEQ-PCDD/Fs	pg-TEQ/g lipid	7.7	1.6	7.5	1.8	7.8	1.4	0.747

*N* number of subjects, *SD* standard deviation, *VND* Vietnamese Dong, p-value: compared between sexes.

*1 missing; **Shown by geometrical means and geometrical standards, *TCDD* 2,3,7,8-tetrachlorodibenzodioxin, *TEQ-PCDD/Fs* toxic equivalency of polychlorinated dibenzo-p-dioxins and polychlorinated dibenzo furans.

A follow-up study was performed when children reached 3 years old. At this time, 50 (21 boys and 29 girls) of 55 (90.9%) children who participated in the 2-year-old survey had their neurodevelopment examined. Five cases who did not participate the survey because of two cases who stayed in the hospital and three cases with their parents who were too busy on the examination day. The number of participants, age, and mean (SD) body size indices of children who had their neurodevelopment examined in the 3-year-old survey are shown in Table 1. The time line of the present study is shown in Supple. data 2.

Informed consent was obtained from all the subjects and their legal guardian (mother’s) involved in the study. All methods were performed in accordance with the relevant guidelines and regulations by the Dong Nai Health Department and the Vietnam Military Medical University. The institutional ethics board for epidemiological studies at Kanazawa Medical University reviewed and approved the study design (No. 187).

### Exposure assessment

Dioxins in maternal breast milk were used as exposure markers because we previously showed a significant association of dioxins between cord blood and breast milk samples^9^.

One month after birth, nurses from community health centers visited the mother’s house and collected approximately 20 ml of breast milk from them. Breast milk samples were frozen, transported to Japan, and levels of 7 congeners of PCDDs and 10 congeners PCDFs were quantified in the High Technology Center at Kanazawa Medical University. After using an EYELA freeze-dryer (FDU-1200; Tokyo-rika Inc., Tokyo, Japan) to dehydrate 10 ml of breast milk, the fat content was extracted using an ASE-200 accelerated solvent extractor (Dionex Co., CA, USA). Next, 13C-labeled 2,3,7,8-substituted PCDDs/Fs (DF-LCS-A40; Wellington Inc., Ontario, Canada) were added as an internal standard. After a series of purification and separation processes, levels of 7 PCDD and 10 PCDF congeners were quantified using a gas chromatograph (HP-6980; Hewlett-Packard, Palo Alto, CA, USA) equipped with a high-resolution mass spectrometer (MStation-JMS700; JEOL, Tokyo, Japan). The concentration of each congener below the detection limit was set by half of the detection limit. TEQ-PCDDs/Fs were calculated as the sum of all values obtained by multiplying each congener concentration by its toxic equivalent factor from WHO 2005-TEF^10^. The established method for analysis was described in detail previously^1,2^.

### Gaze behavior examination

#### Examination method

At 2 years old, the gaze behavior test was performed in community health centers using a Tobii X2-60 Compact eye tracker running at 60 Hz (Tobii Technology, Stockholm, Sweden) to measure the position of both eyes with an infrared camera below the screen. To minimize noise or other factors from outside, which might disturb the attention of the children, the device was set up in a private room with a closed door. Only the examiner and the child with their caregiver stayed inside the room. Children were comfortably seated on the lap of their caregiver in front of the screen of a computer with a distance from their eyes to the screen of approximately 60 cm and free viewing.

Gaze data were extracted and analyzed using Tobii Studio software (Tobii Technology, Stockholm, Sweden). Before extracting data, a default I-VT filter (Tobii Studio software, User manual version 3.3.1, 2015) was applied with a velocity threshold of 30 degrees/s and the minimum fixation duration was set at 100 m.sec. to determine fixation. Two video clips contained a Vietnamese child (a boy for one clip and a girl for the other clip) who was similar in age to the participants. The Vietnamese child engaged in social interaction, such as talking to the camera operator or having a conversation with someone who did not appear on the screen. The video clips were shown for 43 s. to children by the Tobii system. In the interval between the two video clips, one dynamic video of a bear cartoon figure with sound appeared in the center of screen for 2 s. to attract the child’s attention to the screen. Before data recording, children had to achieve successful calibration with a dynamic bear cartoon figure that appeared at the four corners in the top and bottom of the screen and in the middle of the screen, accompanied by sound. Details of this method were described in our previous study^4^.

#### Full frame analysis

The whole frame and facial area were manually set to define areas of interest (AOIs) in each frame for 1336 frames. The total fixation duration in the facial area and in the whole frame were extracted in each frame using Tobii Studio software. By adding up the values in the 1336 frames, the total fixation duration on the screen and facial areas were calculated. The percentage of the total fixation duration on facial areas (% Face) was defined as the ratio of the total fixation duration on facial areas in all frames divided by the total fixation duration on the whole frame in all frames. These ratios were then multiplied by 100 to calculate the percentage of the total fixation duration on facial areas for all stimuli.

#### Analysis for silent and talking scenes

Gaze attention allocation patterns in the eye and mouth regions are different between viewing silent and talking facial scenes. This finding can be explained by the influence of the context of social scenes, particularly auditory speech modulation of gaze attention when viewing a dynamic face^11,12^. We chose five epochs from the videos that lasted for 18.6 s showing talking facial scenes to set up AOIs for gaze analysis. Additionally, we chose five epochs that lasted for 12.4 s. showing a child with a silent face (non-talking), in which both eyes and the mouth of the child were clearly identifiable, to set up AOIs for gaze analysis.

**Figure 1:**
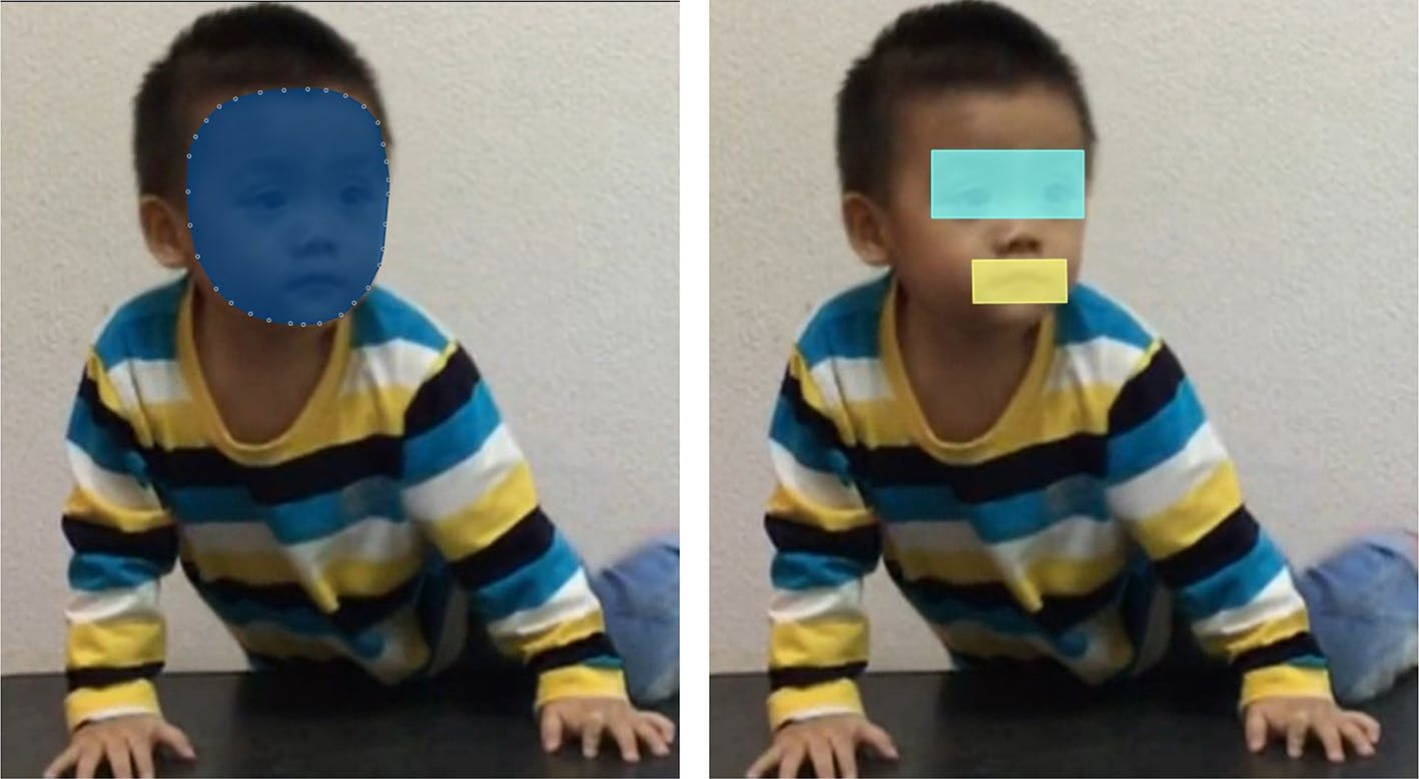
Area of interest set on the face area (**A**), eye and mouth areas (**B**) of a boy in a frame of the video clip.

Similarly, the whole frame, face, mouth, and eye areas were manually set to define AOIs in each frame. Each AOI contained the entire feature for talking and non-taking facial scenes such as eye areas is defined by a single rectangle that included both eyes and eyebrows and mouth areas is defined by a single rectangle that framed the mouth (Fig. 1). There were 557 frames of 5 epochs for talking facial scenes and 370 frames of 5 epochs for silent facial scenes. We extracted the total fixation duration in the whole frame, face, mouth, and eye areas in each frame. We added up the values of the 557 and 370 frames as the total fixation duration on the whole frame, facial areas, mouth areas, and eye areas for viewing talking and silent facial scenes, respectively. The percentages of the total fixation duration on faces (% Face), the mouth (% Mouth), and eye areas (% Eyes) when viewing talking and silent facial scenes were calculated and used as gaze behavior indices for data analysis. Details of this method were described in our previous study^5,13^.

### Neurodevelopmental assessment

At 3 years old, face to face interview mothers or caregivers of children was conducted using full-length parent rating forms (2–5 years) of the Autism Spectrum Rating Scale ([ASRS] MSH, North Tonawanda, NY, USA) to assess child behavior associated with autism spectrum disorder. Before using the ASRS scale for this study, a trial examination was conducted with 15 Vietnamese children to ensure the feasibility and appropriateness of the ASRS for the Vietnamese population. Four main scores, which were the total (TOT) score, the DSM-IV-TR (DSM) score, the Social Communication (SC) score, and the Unusual Behavior (UB) score, were calculated. An increase in TOT and DSM scores is associated with increased autistic behavioral traits. Details of this method were described in our previous study^14^.

The Bayley-III scale (NCS Pearson, Inc., Bloomington, MN, USA) was used to examine general neurodevelopment across five domains of cognition, expressive and receptive language, and fine and gross motor skills. Similarly, we had previously conducted several surveys using The Bayley Scales of Infant and Toddler Development, Third Edition (Bayley-III) scale in groups of Vietnamese infants and children in our previous studies^2,14–16^. Only one examiner who was trained in the previous surveys and blinded to the dioxin exposure levels of the children performed the test to provide reliable scoring. In the present examination, results for ASRS scores were obtained from 50 children and those for general neurodevelopmental scores were obtained from 48 children because 2 children refused to perform some tests.

### Statistical analysis

IBM SPSS version 22.0 (IBM Corp., Armonk, NY, USA) was used for statistical analysis. Children were divided into high and low exposure groups in relation to concentrations of TCDD and TEQ-PCDD/Fs in breast milk. The cut-off value for TCDD (3.5 pg/g lipid) was calculated from the geometric mean × the geometric standard deviation^3^ of TCDD concentrations in breast milk samples from unsprayed areas as we previously reported^15^. Because of a limited number of subjects with TEQ-PCDD/Fs levels higher than the geometric mean × the geometric standard deviation^3^, the cut-off value for TEQ-PCDD/Fs was set at the 75th percentile of TEQ-PCDD/F levels (9.2 pg-TEQ/g lipid) of the subjects.

Gaze behavior indices were converted into z-scores to improve the normal distribution. The Mann–Whitney *U* test was used to compare unadjusted gaze behavior indices between the high and low exposure groups. A general linear model was used to compare adjusted gaze behavior indices after adjusting for maternal years of education. Since smoking have been reported that associated with increase autistic trait and neurodevelopmental disorder in previous studies^17,18^. Therefore, adjusted ASRS and Bayley-III scores were compared between high and low exposure group using a general linear model after adjusting for maternal years of education and family member smoking (yes/no). The p-value ≤ 0.05 was considered statistically significant.

### Ethics approval

All methods were performed in accordance with the relevant guidelines and regulations by the Dong Nai Health Department and the Vietnam Military Medical University. The institutional ethics board for epidemiological studies at Kanazawa Medical University reviewed and approved the study design (No. 187).

### Consent to participate

Informed consent was obtained from all the subjects and their legal guardian(mother's) involved in the study.

## Results

### Comparison of gaze behavior indices between boys and girls

The total fixation duration on the whole screen, % Face, % Mouth, and % Eyes for viewing all, silent, and talking scenes were compared between sexes after adjusting for covariates (Table 2). The adjusted means of the total fixation duration on the screen for viewing all scenes, silent scenes, and talking scenes were higher in girls than in boys. However, only the total fixation duration on the whole screen for talking scenes was significant (*p* = 0.037). Adjusted means of % Mouth were lower and adjusted means of % Eyes were higher in boys for viewing silent and talking scenes compared with girls, but these differences were not significant.

**Table 2:** Comparisons of total and percentage of fixation duration (% FD) on areas of face, mouth, and eyes in the different scenes between boys and girls after adjusting confounding factors.

Scenes	Areas	Units	Boys (N = 24)				Girls (N = 31)				p-value
			Mean	SD	Adj.mean	SE	Mean	SD	Adj.mean	SE	
All	Total	ms	16.1	8.9	16.1	1.9	20.7	8.5	20.6	1.7	0.089
	Face	%	47.5	21.1	46.2	4.0	48.8	17.1	49.8	3.5	0.516
Silent	Total		4.3	2.9	4.4	0.6	5.6	2.7	5.6	0.5	0.145
	Face	%	57.0	26.4	54.7	5.2	58.2	24.0	60.9	4.5	0.380
	Mouth	%	9.5	12.7	9.8	3.2	15.0	16.5	14.7	2.8	0.272
	Eyes	%	20.1	21.7	20.7	4.3	16.3	18.2	15.9	3.7	0.421
Talking	Total	ms	8.9	4.8	8.6	1.0	11.3	4.3	11.5	0.9	0.037
	Face	%	55.9	25.7	54.6	4.9	56.7	20.4	57.7	4.3	0.647
	Mouth	%	10.7	13.3	10.4	3.1	13.9	15.5	14.2	2.7	0.382
	Eyes	%	14.8	15.0	14.5	2.7	12.1	9.5	12.4	2.3	0.571

*N* number of subjects, *SD* standard deviation, *Adj.mean* adjusted mean, *SE* standard error, *FD* fixation duration, *ms* milli-seconds,

Confounding factor maternal education, parity categories, gestational week, and month age at the examination day.

### Associations between perinatal TCDD exposure and gaze behavior

The adjusted means of standardized % Face for all scenes and % Face, % Mouth, and % Eyes for silent and talking scenes were compared between the high and low TCDD exposure groups with a cut-off value of 3.5 pg/g lipid (Table 3). For all participants, there was no significant difference in any gaze indices between high and low TCDD exposure groups. In boys, the means of standardized % Face for all scenes, silent scenes, and talking scenes were lower in the high TCDD group compared with the low TCDD group, but this was only significant for talking scenes (*p* < 0.05). However, in girls, there were no significant differences in the standardized means of any gaze markers for any scenes.

**Table 3:** Comparisons of percentage of fixation duration (% FD) on areas of face, mouth, and eyes in the different scenes between high and low TCDD exposure groups.

Scenes	Areas	TCDD ≥ 3.5 pg/g lipid						TCDD < 3.5 pg/g lipid						MW-U	ANCOVA
		% FD		Z-score				% FD		Z-score					
		Mean	SD	Mean	SD	Adj. mean	SE	Mean	SD	Mean	SD	Adj. mean	SE	*p*-value	*p*-value
All participants		(N = 11)						(N = 44)							
All	Face	42.8	20.4	− 0.259	1.110	− 0.349	0.314	49.6	18.4	0.060	0.980	0.083	0.154	0.385	0.228
Silent	Face	51.5	25.2	− 0.211	1.019	− 0.324	0.300	59.9	23.7	0.110	0.958	0.139	0.147	0.302	0.176
	Mouth	8.3	15.9	− 0.309	0.968	− 0.308	0.311	13.7	14.9	0.099	1.004	0.098	0.152	0.131	0.252
	Eyes	13.9	16.1	− 0.167	0.875	− 0.187	0.319	19.0	20.5	0.065	1.038	0.069	0.156	0.548	0.480
Talking	Face	50.7	27.8	− 0.218	1.265	− 0.319	0.312	57.7	21.3	0.027	0.935	0.052	0.153	0.397	0.296
	Mouth	11.1	9.6	− 0.194	1.035	− 0.169	0.312	13.1	14.3	0.035	0.975	0.029	0.153	0.309	0.577
	Eyes	11.1	9.6	− 0.167	0.913	− 0.270	0.311	13.9	12.8	0.030	1.022	0.056	0.152	0.565	0.356
Boys		(N = 4)						(N = 20)							
All	Face	30.3	16.4	− 0.854	0.789	− 0.878	0.485	51.0	20.5	0.143	0.987	0.148	0.213	0.056	0.063
Silent	Face	34.9	16.8	− 0.823	0.645	− 0.837	0.492	61.4	26.0	0.193	0.999	0.196	0.217	0.081	0.066
	Mouth	3.3	6.5	− 0.500	0.525	− 0.501	0.533	10.8	13.4	0.089	1.103	0.089	0.244	0.273	0.326
	Eyes	12.5	14.1	− 0.326	0.655	− 0.324	0.523	21.6	22.9	0.151	1.070	0.150	0.240	0.525	0.420
Talking	Face	33.4	23.9	− 0.917	0.933	− 0.938	0.479	60.4	24.1	0.140	0.943	0.145	0.210	0.045	**0.047**
	Mouth	1.3	1.6	− 0.748	0.119	− 0.735	0.478	12.6	13.8	0.125	1.016	0.122	0.219	0.627	0.119
	Eyes	10.5	10.2	− 0.270	0.686	− 0.286	0.533	15.7	15.9	0.089	1.102	0.093	0.244	0.115	0.525
Girls		(N = 7)						(N = 24)							
All	Face	49.9	19.8	0.081	1.171	0.031	0.429	48.4	16.7	− 0.008	0.991	0.006	0.219	0.562	0.960
Silent	Face	60.9	25.1	0.138	1.063	− 0.059	0.390	58.7	22.1	0.041	0.937	0.099	0.199	0.964	0.729
	Mouth	11.2	19.4	− 0.199	1.177	− 0.042	0.414	16.1	15.9	0.095	0.965	0.049	0.212	0.302	0.852
	Eyes	14.8	18.2	− 0.077	1.017	− 0.160	0.427	16.8	18.5	0.037	1.034	0.061	0.218	0.824	0.660
Talking	Face	60.5	26.3	0.181	1.313	0.173	0.427	55.5	18.8	− 0.068	0.937	-0.066	0.218	0.370	0.634
	Mouth	15.4	18.6	0.123	1.207	0.125	0.425	13.5	14.9	0.001	0.964	0.000	0.217	0.894	0.802
	Eyes	11.4	10.1	− 0.108	1.068	− 0.206	0.420	12.3	9.5	− 0.012	0.999	0.016	0.214	0.929	0.652

Significant values are in bold.

*N* number of subjects, *SD* standard deviation, *Adj.mean* adjusted mean for maternal education, and gender for all participants and for maternal education for each sex, *SE* standard error, *FD* fixation duration, *MW-U* Mann–Whitney *U* test.

### Associations between perinatal TEQ-PCDD/Fs exposure and gaze behavior

The adjusted means of standardized % Face for all scenes and % Face, % Mouth, and % Eyes for silent and talking scenes were compared between the high and low TEQ-PCDD/Fs exposure groups with a cut-off value of 9.2 pg-TEQ/g lipid (Table 4). No significant difference was found in the standardized means of any gaze marker for all participants. Similarly, in boys, there were no significant differences in the standardized means of any gaze markers for any scenes. However, in girls, the adjusted mean of standardized % Eyes for talking scenes was significantly lower in the high TEQ-PCDD/Fs exposure group than in the low TEQ-PCDD/Fs exposure group (*p* < 0.05). These results suggest a shorter fixation duration of gaze on the eye area of a talking person in girls who are exposed to high TEQ-PCDD/Fs.

**Table 4:** Comparisons of percentage of fixation duration (% FD) on areas of face, mouth, and eyes in the different scenes between high and low TEQ-PCDD/Fs exposure groups.

Scenes	Areas	TEQ-PCDD/Fs ≥ 9.2 pg-TEQ/g lipid						TEQ-PCDD/Fs < 9.2 pg-TEQ/g lipid						MW-U	ANCOVA
		% FD		Z-score				% FD		Z-score					
		Mean	SD	Mean	SD	Adj mean	SE	Mean	SD	Mean	SD	Adj mean	SE	p-value	p-value
All participants		(N = 15						(N = 40)							
All	Face	48.2	16.9	− 0.024	0.933	− 0.174	0.294	48.3	19.7	0.004	1.042	0.060	0.169	0.947	0.515
Silent	Face	57.7	18.9	0.034	0.764	− 0.159	0.281	58.4	25.9	0.051	1.044	0.123	0.161	0.762	0.414
	Mouth	8.8	15.1	− 0.263	0.905	− 0.226	0.292	14.0	15.0	0.122	1.027	0.108	0.168	0.099	0.352
	Eyes	17.0	18.2	− 0.047	0.910	− 0.098	0.297	18.3	20.5	0.043	1.047	0.062	0.170	0.932	0.660
Talking	Face	59.4	23.0	0.095	1.061	0.011	0.295	55.2	22.6	− 0.066	0.988	− 0.035	0.169	0.664	0.899
	Mouth	14.4	15.7	0.106	1.046	0.202	0.289	11.8	14.2	− 0.054	0.967	− 0.090	0.166	0.507	0.409
	Eyes	11.0	10.5	-0.246	0.919	− 0.453	0.285	14.2	12.8	0.079	1.021	0.157	0.163	0.526	0.084
Boys		(N = 7)						(N = 17)							
All	Face	51.1	16.1	0.15	0.77	0.07	0.40	46.8	22.7	− 0.09	1.11	− 0.06	0.25	0.701	0.798
Silent	Face	51.8	17.0	− 0.17	0.65	− 0.24	0.40	58.1	29.1	0.10	1.14	0.13	0.26	0.615	0.452
	Mouth	3.1	5.5	− 0.51	0.44	− 0.56	0.39	12.0	13.6	0.21	1.12	0.23	0.25	0.085	0.107
	Eyes	21.4	19.9	0.09	0.93	0.11	0.41	18.8	22.6	0.00	1.07	− 0.01	0.26	0.615	0.824
Talking	Face	63.4	21.1	0.26	0.82	0.20	0.40	55.0	26.7	− 0.16	1.07	− 0.13	0.25	0.458	0.502
	Mouth	9.4	13.6	− 0.16	1.00	− 0.11	0.39	13.3	14.1	− 0.02	0.99	− 0.04	0.25	0.929	0.893
	Eyes	14.1	10.6	− 0.03	0.71	− 0.10	0.41	14.7	16.0	0.04	1.14	0.07	0.26	0.836	0.728
Girls		(N = 8)						(N = 23)							
All	Face	45.6	18.3	− 0.18	1.08	− 0.50	0.47	49.6	16.7	0.08	1.01	0.19	0.24	0.623	0.247
Silent	Face	62.7	20.1	0.21	0.85	− 0.14	0.43	56.0	24.8	0.01	0.99	0.13	0.22	0.685	0.613
	Mouth	13.8	19.2	− 0.04	1.16	0.37	0.46	14.8	15.9	0.05	0.97	− 0.09	0.23	0.654	0.422
	Eyes	13.2	16.8	− 0.17	0.94	− 0.47	0.47	17.1	18.5	0.07	1.05	0.18	0.24	0.623	0.271
Talking	Face	55.9	25.5	− 0.05	1.27	− 0.20	0.48	57.1	18.2	0.00	0.94	0.05	0.24	0.949	0.671
	Mouth	18.7	16.9	0.34	1.10	0.58	0.46	11.3	14.8	− 0.08	0.97	− 0.16	0.23	0.160	0.208
	Eyes	8.3	10.2	− 0.44	1.08	− 0.99	0.43	13.9	8.8	0.11	0.95	0.30	0.22	0.204	**0.021**

Significant values are in bold.

*N* number of subjects, *SD* standard deviation, *Adj.mean* adjusted mean for maternal education, and gender for all participants and for maternal education for each sex, *SE* standard error, *FD* fixation duration, *MW-U* Mann–Whitney *U* test, *TEQ-PCDD/Fs* toxic equivalency of polychlorinated dibenzo-p-dioxins and polychlorinated dibenzo furans.

### Comparisons of autistic behavior indicated by the ASRS scores between high and low dioxin exposure groups

Autistic behavior in the children was examined when they reached 3 years old by the ASRS*.* Adjusted mean scores of TOT and the three subscales DSM, SC, and UB were compared between the high and low TCDD exposure groups after adjusting for maternal years of education and family member smoking status (Table 5). High TCDD group showed significantly decrease in the adjusted mean TOT, DSMT, and SCT scores when compared with the low TCDD exposure group for all participants (*p* < 0.05). Similarly, the adjusted mean DSM score was significantly higher in the high TCDD group compared with the low TCDD group in boys (*p* < 0.05). In girls, the adjusted mean TOT and DSM scores were significantly higher in the high TCDD group compared with the low TCDD group. There were no significant differences in ASRS scale scores between the high and low TEQ-PCDD/Fs exposure groups in either sex (data not shown). These findings suggest that perinatal TCDD exposure increases autistic traits in both sexes.

**Table 5:** Comparison of ASRS scores examined at 3 years of age between high and low TCDD exposure groups.

	TCDD ≥ 3.5 pg/g lipid				TCDD < 3.5 pg/g lipid				p-value
	Mean	SD	Adj mean	SE	Mean	SD	Adj mean	SE	
All	(N = 11)				(N = 39)				
TOT	60.4	6.3	60.5	1.7	56.0	4.9	56.0	0.9	**0.020**
DSMT	61.4	7.8	61.2	1.7	55.5	4.7	55.5	0.9	**0.006**
SCT	59.2	10.3	58.9	2.2	53.7	5.7	53.8	1.1	**0.050**
UBT	58.7	2.9	59.4	1.5	56.8	5.3	56.7	0.8	0.120
Boys	(N = 4)				(N = 17)				
TOT	61.0	5.4	61.0	2.3	56.9	4.2	56.9	1.1	0.125
DSMT	62.8	7.1	62.9	2.6	56.7	4.8	56.6	1.2	**0.043**
SCT	60.3	8.1	60.0	2.8	54.2	5.4	54.3	1.4	0.083
UBT	58.8	2.4	59.0	2.1	57.9	4.4	57.8	1.0	0.637
Girls	(N = 7)				(N = 22)				
TOT	60.0	7.1	61.1	2.4	55.3	5.3	55.0	1.3	**0.036**
DSMT	60.6	8.5	60.9	2.4	54.6	4.6	54.5	1.3	**0.030**
SCT	58.6	12.0	59.1	3.3	53.2	6.0	53.1	1.8	0.130
UBT	56.1	5.9	60.2	2.0	58.7	3.4	55.6	1.1	0.061

Significant values are in bold.

*N* number of subjects, *SD* standard deviation, *Adj.mean* adjusted mean for maternal education, gender, and family member smoking for all participants and for maternal years of education, and family member smoking for each sex, *SE* standard error, *TOT* total score, *DSM* DSM-IV-TR score, *SCT* Social Communication score, *UBT* Unusual Behavior score, *TCDD* 2,3,7,8-tetrachlorodibenzodioxin.

### Comparisons of general neurodevelopment indicated by the Bayley-III scores between high and low dioxin exposure groups

The general neurodevelopmental status of children at 3 years old was also examined using the Bayley-III. We compared these neurodevelopmental scale scores between the high and low TCDD groups after adjusting for maternal years of education and family member smoking status (Table 6). The adjusted mean score was significantly lower in the high TCDD group than in the low TCDD group only for the gross motor scale in girls. There were no significant differences in Bayley III scores between the high and low TEQ-PCDD/Fs groups in either sex (data not shown).

**Table 6:** Comparisons of Bayley-III scores at 3 years of age between high and low TCDD exposure groups.

	TCDD ≥ 3.5 pg/g lipid				TCDD < 3.5 pg/g lipid				p-value
	Mean	SD	Adj mean	SE	Mean	SD	Adj mean	SE	
All	(N = 9)				(N = 39)				
Cognition	91.1	4.9	91.3	2.2	91.4	6.6	91.3	1.0	0.998
Composite language	94.8	12.2	95.8	3.4	94.4	9.6	94.1	1.6	0.656
Expressive language	8.1	1.7	8.4	0.5	8.7	1.6	8.6	0.3	0.627
Receptive language	10.1	2.6	10.2	0.7	9.3	2.0	9.3	0.3	0.241
Composite motor	99.4	7.4	99.7	4.8	106.6	15.9	106.5	2.2	0.211
Fine motor	9.9	2.1	9.9	0.9	10.2	2.8	10.2	0.4	0.769
Gross motor	9.9	1.5	10.0	1.0	12.0	3.2	12.0	0.5	0.077
Boys	(N = 3)				(N = 17)				
Cognition	90.0	0.0	89.2	3.9	90.3	7.0	90.4	1.6	0.788
Composite language	97.0	3.0	95.8	5.1	91.3	9.8	91.5	2.1	0.415
Expressive language	8.3	0.6	8.3	1.0	8.5	1.9	8.5	0.4	0.836
Receptive language	10.7	1.2	10.3	1.0	8.5	1.8	8.6	0.4	0.111
Composite motor	101.0	3.5	98.7	8.4	100.3	15.5	100.7	3.4	0.831
Fine motor	9.3	1.5	9.0	1.6	9.5	2.9	9.6	0.6	0.767
Gross motor	11.0	1.7	10.6	1.5	10.6	2.7	10.6	0.6	0.968
Girls	(N = 6)				(N = 22)				
Cognition	91.7	6.1	92.1	2.9	92.3	6.3	92.2	1.4	0.989
Composite language	93.7	15.2	94.4	4.7	96.8	8.8	96.6	2.3	0.695
Expressive language	8.0	2.1	8.2	0.7	8.9	1.3	8.8	0.3	0.451
Receptive language	9.8	3.1	9.9	1.0	10.0	1.9	10.0	0.5	0.945
Composite motor	98.7	9.0	99.1	6.3	111.7	14.7	111.6	3.1	0.097
Fine motor	10.2	2.5	10.0	1.2	10.7	2.7	10.8	0.6	0.561
Gross motor	9.3	1.2	13.1	0.7	13.1	3.2	13.1	0.7	**0.038**

Significant values are in bold.

*SD* standard deviation, *SE* Standard error, *Adj.mean* adjusted mean for maternal education, gender, and family member smoking for all participants and for maternal years of education, and family member smoking for each sex; N: number of subjects, *TEQ-PCDD/Fs* Toxic equivalent of polychlorinated dibenzo-p-dioxins and polychlorinated dibenzo furans.

## Discussion

In the present study, we found significant effects of perinatal dioxin exposure on gaze behavior only when viewing conversation scenes in 2-year-old Vietnamese children with sex differences in affected gaze areas and exposure markers, lower % Face in the high TCDD group for boys and lower % Eyes in the high TEQ-PCDD/Fs group for girls. A few studies have reported effects of dioxin on gaze behavior in previous reports. In Japan, Doi et al.^19^ investigated prenatal exposure to 17 polychlorinated biphenyl (PCB) congeners on gaze behavior of Japanese infants when viewing biological motion stimuli displayed by point-light. They reported that no significant difference in the fixation duration on inverted and upright biological motions between the high and low PCB#118 exposure groups. However, infants exposed to high PCB#118 preferred to fix their gaze on inverted biological stimuli rather than reducing attention to upright biological motion^19^. These results from a Japanese cohort are consistent with our results, albeit different stimuli were used between our study and the previous study from Japan. Taken together, these findings suggest that there are limited effects of perinatal dioxin and PCB exposure on gaze behavior of infants when viewing only biological motion, including upright facial motion.

In children aged 3 years from our larger Bien Hoa birth cohort 2012, we found decreased % Face associated TCDD exposure in girls, although we used static facial pictures of children for stimuli^5,13^. However, dynamic facial stimuli are likely to have a more natural form, and motion and higher social complexity than point-light stimuli or static pictures^7,20^. In addition, Schilbach et al. suggested that the use of more complex and ecologically valid social stimuli was useful to test social abilities^21^. To the best of our knowledge, the present study is the first to report effects of perinatal dioxin exposure on gaze behavior when viewing dynamic facial stimuli, particularly talking facial scenes.

Prenatal PCB exposure has been reported to affect gaze behavior in infants^19^. We previously reported concentrations of dioxin-like PCBs in breast milk samples that were collected from a birth cohort in Bien Hoa City in 2012^1^. We found that dioxin-like PCB concentrations were low and they were even lower compared with concentrations in samples that were collected from unsprayed areas. These findings suggest that the contribution of TEQ-dl-PCBs to the TEQ is not high and that TEQ-PCDDs/Fs may be an indicator of dioxin toxicity in this contaminated area.

In our previous study of the present subjects, we found that perinatal TCDD and TEQ-PCDD/F exposure affected neonatal neuronal activity and functional connectivity as indicated by relative EEG power and coherence between various brain areas during active and quiet sleep^3,4^. This exposure might also be associated with poorer language development^3^ and less fixation on facial areas when they reach 2 years old^4^. However, we did not stratify by sex in these previous studies. At that time, we used only the fixation duration on facial areas, including the fixation duration on eye areas when viewing all stimuli as a gaze behavior index. Our findings suggest that altered brain development before birth induced by TCDD and TEQ-PCDD/F exposure affects gaze behavior when viewing dynamic facial stimuli.

In this study, we also found that the adjusted mean DSM score for boys and the adjusted mean TOT and DSM scores for girls were significantly higher in the high TCDD exposure group than in the low TCDD exposure group at 3 years of age. Using the same ASRS questionnaires in another cohort of dioxin contamination in Da Nang, Vietnam, we reported that increased perinatal exposure to TCDD levels was associated with increased TOT scores at 3 years old in boys and girls^14^. This association were more pronounced in boys than in girls. These results indicate that perinatal TCDD exposure increases autistic trait behavior in early childhood in Bien Hoa and Da Nang Cities in Vietnam. Reduced facial attention as shown by a shorter fixation duration on facial areas when viewing talking facial scenes has also been reported in autistic children^6,7^. Taken together, these findings suggest that perinatal TCDD exposure increases autistic trait behavior in boys, which might be associated with less attention on facial areas when viewing the face while talking.

According our previous report that there was no significant effect of TEQ-PCDD/Fs on autistic behavioral traits in 3-year-old children in Da Nang^14^, which is consistent with findings in the present study. We did not find any significant association between perinatal TEQ-PCDD/Fs exposure and ASRS scores or neurodevelopmental scores as indicated by Bayley-III scores examined at 3 years old in boys and girls (unpublished data). In our previous studies where we recorded EEG signals during active and quiet sleep in the present subjects, we reported that TEQ-PCDD/Fs levels were significantly associated with relative EEG power or coherence in fewer brain regions than with TCDD levels^3,4^. In a neurodevelopmental study in 2-year-old children in Bien Hoa City including the present subjects, increased perinatal TCDD exposure levels were associated with a decreased expressive language score. However, there were no significant effects of perinatal TEQ-PCDD/Fs exposure on Bayley-III scores in boys or girls^2^. These results indicate that adverse effects of perinatal dioxin exposure originating from Agent Orange on neurodevelopment of children are mainly characterized by effects of TCDD exposure.

In the present study, we only found a decreased percentage of the total fixation duration on eye areas when viewing talking in girls who were exposed to high TEQ-PCDD/Fs levels. This finding suggested that eye contact was reduced when viewing talking in girls who were exposed to high TEQ-PCDD/Fs levels. In previous studies in Da Nang city, Pham et al. (2015) reported that increased perinatal TEQ-PCDD/Fs exposure levels were associated with decreased social emotional scores in infants at 1 year old^22^, expressive language in 3 years-old-girls^14^, increased hyperactivity score in girls at 8 years of age^23^. In the Netherlands, alterations in visual processing and cognition induced by perinatal dioxin exposure as indicated by TEQ-PCCD/Fs levels in maternal breast milk were found in school-aged children^24^. These results suggest that perinatal TEQ-PCDD/Fs exposure might affect specific domains of neurodevelopment, such as communication and social cognition in girls. Additionally, it was also reported that children with hyperactivity often show social interaction impairments related to processing of other’s eye gaze such as lack of attention to the eye region of faces appears^25^. Therefore, follow-up of these children to carefully examine higher cognitive function and symptoms of attention deficit hyperactivity disorder in childhood is necessary.

Sex-specific effects of perinatal dioxin exposure on neurodevelopment or physical growth have been reported in epidemiological studies conducted not only in Vietnam, but also in Japan^26,27^ and The Netherlands^28^. A previous study investigated dioxin and PCB exposure on neurodevelopment of infants in a Hokkaido cohort in Japan and reported that adverse effects of perinatal dioxin exposure were more pronounced in boys than in girls^27^. In Rotterdam, The Netherlands, adverse effects of perinatal exposure to TEQ-PCDD/Fs on playing behavior were found only in boys and not in girls^28^. In Da Nang City, Vietnam, effects of perinatal exposure to TCDD and TEQ-PCDD/Fs on child neurodevelopment were found only in boys at 3, 5, and 8 years^14,16,29,30^. In the Bien Hoa region, we reported that perinatal exposure to high TCDD levels decreased expressive language and gross motor scores only in 2-year-old-boys^2^. Similarly, in the current study, a change in the percentage of the total fixation duration on face areas when viewing talking scenes was found only in boys and not in girls with perinatal exposure to TCDD. Our findings in Vietnam also suggest that boys are more susceptible to toxicity of perinatal TCDD exposure on neurodevelopment than girls.

However, in a 3-year-follow-up study for children from Bien Hoa birth cohort 2012, we found a decreased percentage of the total fixation duration on facial areas when viewing static facial images was also found in 3-year-old-girls who were exposed to high TCDD levels (3.5 pg/g lipid)^5,13^. Additionally, in the Da Nang cohort, we found effects of perinatal TCDD exposure on neurodevelopment in girls at 3 and 8 years old, which resulted in increased autistic behavior at 3 years old^14^ and feminine gaze behavior at 8 years old^31^. However, no effect on learning ability was found in these girls at 8 years old^30^. These findings suggest that neurodevelopmental effects induced by perinatal TCDD exposure in girls may be detectable in different domains of neurodevelopment and at a different age from boys.

In the present study, we found that perinatal TEQ-PCDD/Fs exposure decreased attention to the eyes when viewing talking scenes in girls. Seeing the face, including eye contact, can help children to improve their learning ability and social skills, leading to good neurodevelopment in the future^32,33^. A developmental milestone from 2 years can predict development in children and adulthood^34,35^ or is associated with psychiatric disorders, including neurodevelopmental disorders, such as autism diagnosed later in life^36^. Therefore, a follow-up study of these children is necessary until school age to clarify the association between dioxin exposure and neurodevelopment, especially language ability and social communication skills.

### Limitations

In this study, we used eye tracking, which is an objective tool for assessment of gaze behavior. We found an adverse effect of perinatal dioxin exposure on the gaze pattern when viewing dynamic facial stimuli, particularly viewing talking facial scenes in 2-year-old Vietnamese children. However, we should interpret the present data within the context of several limitations. We used two videos of a Vietnamese boy in one video and a Vietnamese girl in the other who were interacting with the camera operator as stimuli to attract children to engage in the gaze test. However, these stimuli appeared to attract visual attention more effectively in girls compared with boys. Furthermore, this study had a relatively small sample size which made dose–response analysis difficult and did not have a control group of participants in non-exposed areas. Therefore, further epidemiological studies with a sufficient number of subjects and un-exposed controls with gaze behavior measurement when viewing a dynamic face are necessary to clarify this issue.

## Conclusion

Perinatal dioxin exposure indicated by TCDD and TEQ-PCDD/Fs levels in breast milk reduced gaze behavior in 2-year-old children when viewing dynamic facial stimuli, particularly talking scenes, reflects their social communication ability. However, the effects of TCDD exposure on gaze behavior was more specific in boys than in girls, and TEQ-PCDD/Fs exposure was associated with decreased gaze behavior in girls. Furthermore, children with high TCDD levels in both sexes show increased autistic traits after 1 year of follow-up. These results suggest that poor gaze behavior, particularly in the face of children while talking, may predict increased autistic traits in early childhood.

### Supplementary Information

Supplementary Information.

## Data Availability

The datasets used and analysed during the current study available from the corresponding author on reasonable request.

## Abbreviations

TCDD: 2,3,7,8-Tetrachlorodibenzo-p-dioxin
TEQ: Toxic equivalency
PCDDs: Polychlorinated dibenzo-p-dioxins
PCDFs: Polychlorinated dibenzo-p-dioxins
PCDD/Fs: Polychlorinated dibenzo-p-dioxins/dibenzofurans
FD: Fixation duration
AOI: Areas of interest
Bayley-III: The Bayley scales of infant and toddler development, third edition
ASRS: The Autism spectrum rating scale
FD: Fixation duration
