# Supplementary material for: Impacts of perinatal dioxin exposure on gaze behavior in 2-year-old children in the largest dioxin-contaminated area in Vietnam
Source: Sci Rep. 2023 Nov 24;13:20679. doi: 10.1038/s41598-023-47893-0 (PMC10673870; doi:10.1038/s41598-023-47893-0)
Supplement: Supplementary file 1 — Supplementary Information. [file 41598_2023_47893_MOESM1_ESM.docx]

Supple. Data 1. Comparisons of breast dioxin concentration levels between the groups with and without participants.

|  | Participants (N=55) | | Non-participants (N=12) | |  |
| --- | --- | --- | --- | --- | --- |
|  |  |  |  |  |  |
|  | GM | GSD | GM | GSD | P-value |
| PCDD congeners |  |  |  |  |  |
| 2,3,7,8-TetraCDD | 2.1 | 2.2 | 1.6 | 1.9 | 0.276 |
| 1,2,3,7,8-PentaCDD | 2.6 | 1.4 | 2.2 | 1.6 | 0.196 |
| 1,2,3,4,7,8-HexaCDD | 1.2 | 1.6 | 1.1 | 1.4 | 0.305 |
| 1,2,3,6,7,8-HexaCDD | 3.6 | 1.6 | 3.7 | 1.8 | 0.838 |
| 1,2,3,7,8,9-HexaCDD | 1.5 | 1.6 | 1.3 | 1.9 | 0.295 |
| 1,2,3,4,6,7,8-HeptaCDD | 7.3 | 1.7 | 6.6 | 2.3 | 0.553 |
| OctaCDD | 61.4 | 1.8 | 60.4 | 2.4 | 0.936 |
| PCDF congeners |  |  |  |  |  |
| 2,3,7,8-TetraCDF | 0.7 | 1.7 | 0.5 | 1.7 | 0.214 |
| 1,2,3,7,8-PentaCDF | 0.7 | 1.8 | 0.6 | 1.8 | 0.317 |
| 2,3,4,7,8-PentaCDF | 2.9 | 1.5 | 2.8 | 1.4 | 0.778 |
| 1,2,3,4,7,8-HexaCDF | 5.1 | 1.6 | 4.0 | 1.6 | 0.123 |
| 1,2,3,6,7,8-HexaCDF | 2.6 | 1.6 | 2.3 | 1.4 | 0.414 |
| 1,2,3,7,8,9-HexaCDF | 0.5 | 1.8 | 0.4 | 1.9 | 0.336 |
| 2,3,4,6,7,8-HexaCDF | 0.7 | 1.6 | 0.5 | 1.6 | 0.219 |
| 1,2,3,4,6,7,8-HeptaCDF | 2.3 | 1.6 | 2.1 | 2.0 | 0.498 |
| 1,2,3,4,7,8,9-HeptaCDF | 0.5 | 1.8 | 0.5 | 1.8 | 0.771 |
| OctaCDF | 1.7 | 2.2 | 1.4 | 1.9 | 0.395 |
| TEQs |  |  |  |  |  |
| PCDDs | 5.7 | 1.7 | 4.8 | 1.5 | 0.267 |
| PCDFs | 1.9 | 1.4 | 1.8 | 1.2 | 0.360 |
| PCDD/Fs | 7.8 | 1.6 | 6.7 | 1.3 | 0.275 |

N: number of subject; GM: geometrical mean; GSD: geometrical standard deviation;

TEQ: toxic equivalency; PCDDD: polychlorinated dibenzo-p-dioxins; PCDF: polychlorinated dibenzo furans; PCDD/Fs polychlorinated dibenzo-p-dioxins and polychlorinated dibenzo furans;

p-value: compared between two groups by the independent samples t-test

Supple. data 2. Time line of the present study.

**Dioxin contaminated in Bien Hoa**

The main source is the result of the storage, loading, spillage, and handling of Agent Orange and other toxic herbicides during the US-Viet Nam war, especially between 1965 and 1971**.**

High dioxin concentration levels in soil and sediment samples collected in and around the Bien Hoa airbase in 2008, 2010 (Hatfield Consultants, 2011).

Reduced gaze behaviour at 2 years

Increased autistic trait behaviour at 3 years

Infants are exposed to dioxins from mothers via cord blood and breast feeding

High dioxin concentration levels in maternal breast milk in residents living nearby the Bien Hoa airbase collected in 2015
